# Infective (“Secondary”) Hemorrhages from War Wounds, a Clinical and Pathological Study

**Published:** 1919

**Authors:** 


### SURGERY

---

***Infective (" Secondary ") Hemorrhages from War Wounds, a Clinical and Pathological Study.*** By HAROLD NEUHOF, Captain, M. C., and FORDYCE BARKER ST. JOHN, Captain M. C. U. S. Base Hospital N° 2 (N° 1 [Presbyterian, U. S. A.] General Hospital B. E. F.).

Conflicting views are held upon the cause and treatment of infective hemorrhage, and its pathology has not been described. In a year's experience at the Base Hospital the condition has been a serious and not infrequent complication (95 operations for infective hemorrhage in a total of 2332 operations). The term " infective " rather than " secondary hemorrhage " is employed because the study demonstrates that infection was the cause of the hemorrhages that were encountered. Secondary hemorrhage is a loosely used expression for all but primary hemorrhages consecutive or secondary to a variety of causes.

The average time for the onset of bleeding was 12.8 days, but ranged from five to forty-seven days. The danger from infective hemorrhage is over only when infection has ceased. Recurrence of bleeding is not uncommon. The slighter hemorrhages cannot be ignored, for the amount of bleeding is absolutely no guide to the size of the vessel involved or the extent of the lesion. None of the wounds from which hemorrhages arose were in a satisfactory condition either clinically or bacteriologically. Those that appeared only slightly infected proved to be more extensively so in the depths, either at a revisional operation or one for the control of the hemorrhage. Infection along the sheaths of the vessels was always present, but usually did not extend far beyond the site of rupture in the artery. The latter, commonly some little distance from the confines of the wound, ordinarily involved less than half the circumference of the vessel.

Microscopic examinations of removed specimens show artery and accompanying vein or veins embedded in infected granulation tissue. There is considerable edema of the arterial wall, which is otherwise usually well preserved to the immediate vicinity of rupture. Here there is degeneration, with round and polynuclear cell invasion, merging into massive necrosis. The intima is often spread over the abrupt ending of the muscularis. A recent thrombus is often present. In a single instance this was partly organized and extensive, and the microscopic picture was that of a dissecting aneurism. In this case there was slight bleeding that did not recur, the patient dying from another cause. From the examination of their material the authors believe that the pathological picture of infective hemorrhage before rupture is this type of dissecting false aneurism. Stains for bacteria showed various organisms in varying numbers grouped characteristically in the periphery of the thrombus, at and in the intima and adventitia, in the necrotic media, but strikingly few or none in the remainder of the muscularis.

Involvement of the accompanying vein in cases of infective hemorrhage is frequent and often fatal. The pathological alterations range from the slighter grades of phlebitis with infective thrombus to extensive suppurative thrombosis. In the great majority of postmortem examinations of patients who have died of infective hemorrhage, systemic infection with pyemic foci have been found, the source being the infected venous trunks accompanying the diseased arteries.

The arterial pathology in infective hemorrhage consecutive to war wounds was identical, in two cases observed by the authors, with that of so-called " secondary hemorrhage " complicating ordinary infected wounds of civil life. They believe this is additional evidence that a primary wound of an artery need not be invoked to account for " secondary hemorrhage ". While not denying that an untreated arterial wound with or without added infection may lead to such hemorrhages, they consider that this is not the usual cause. The absence of any microscopic signs of injury, the invariable presence of infection, the site of the arterial rupture (usually at some distance from the wound), and the equal or greater extent of infection in the vein accompanying the artery, all indicate that infection alone is the common cause.

The authors discuss the operative treatment of infected wounds only in so far as the vessels are concerned. They believe that special attention should be paid to vascular structures if they are in immediate anatomic relationship to an infected tract. Under such circumstances they should be adequately exposed, and whatever form of chemical sterilization is planned should be applied directly to their sheaths. The operative exposure of the vessels may reveal erosion of the artery and thrombosis of the vein at the time when the best treatment for these lesions may be applied.

Treatment for the hemorrhage itself was conservative in the earlier cases, especially for the slighter bleedings, with unfortunate results. It was found to be dangerous, even fatal, to temporize by wound packing, etc., occurrence of more serious bleeding and further extension of infection being the usual sequel. Amputation is indicated for most of the infective hemorrhages from the popliteal or posterior tibial arteries, particularly when fracture co-exists. Ligation of the main arteries at the level of amputation is insufficient when amputation is done through an infected field. Whenever the patient's condition permits the additional time, exposure and ligation should be done at a non-infected level; the accompanying vein should be examined for the presence of a phlebitis and treated accordingly.

Concerning the treatment of the many cases in which amputation is not indicated, hemorrhage recurred frequently after operations consisting in ligation or resection of the artery in the wound. The source of bleeding could not always be discovered, the satisfactory placement of ligatures was difficult, especially for deeply situated or friable vessels, the infected tract along the vessels was not laid open, and the condition of the vein could not be determined. The authors therefore advocate what they term a " surgical approach " to the artery, through a separate incision whenever feasible, and its double ligation and resection beyond the rupture and the infected tract. They have not reached a definite conclusion as to the treatment of the accompanying vein (when it is an important trunk and shows no visible involvement. In two of their cases aspiration of such veins withdrew fluid blood, yet infected parietal thrombi were present. During the war observations on ligation of veins accompanying damaged arteries appear to have established not only the innocuousness of the vein ligation but as well a resulting reduction in the frequency of gangrene. For this reason as well as for the probability of vein involvement in infective hemorrhage, the authors incline towards ligation or resection of the accompanying vein, even though it be an important trunk without visible evidence of thrombosis.

